# Association of Platelet to lymphocyte ratio with non-culprit atherosclerotic plaque vulnerability in patients with acute coronary syndrome: an optical coherence tomography study

**DOI:** 10.1186/s12872-017-0618-y

**Published:** 2017-07-03

**Authors:** Xuedong Wang, Zulong Xie, Xinxin Liu, Xingtao Huang, Jiale Lin, Dan Huang, Bo Yu, Jingbo Hou

**Affiliations:** 1 Department of Cardiology, The Second Affiliated Hospital of Harbin Medical University, Harbin, 150001 China; 2 Key Laboratory of Myocardial Ischemia, Ministry of Education, Harbin Medical University, Harbin, 150001 China; 3 Department of Cardiology, The Second Affiliated Hospital of Chongqing Medical University, Chongqing, 400010 China

**Keywords:** Platelet to lymphocyte ratio, Atherosclerosis, Plaque vulnerability, Optical coherence tomography

## Abstract

**Background:**

The platelet to lymphocyte ratio (PLR), an indirect inflammatory biomarker, has been recently demonstrated to be associated with severity of coronary artery disease. In the present study, we sought to investigate whether PLR is associated with vulnerable plaque characteristics of non-culprit lesions in patients with acute coronary syndrome (ACS).

**Methods:**

The patients in our study were divided into two groups (high PLR group and low PLR group). A total of 119 non-culprit plaques from 71 patients with ACS were assessed by optical coherence tomography (OCT).

**Results:**

The non-culprit plaques in high PLR group exhibited thinner fibrous cap thickness (FCT) (88.60 ± 44.70 vs. 119.28 ± 50.22 μm, *P* = 0.001), greater maximum lipid arc (271.73 ± 71.66 vs. 240.60 ± 76.69°, *P* = 0.027) and increased incidence of thin-cap fibroatheroma (TCFA) (34.0% vs. 15.9%, *P* = 0.022) compared with those in low PLR group. Meanwhile, PLR was negatively associated with FCT (*r* = −0.329, *P* < 0.001). Furthermore, multivariate regression analysis showed that PLR [OR: 1.023 (95% CI: 1.005–1.041), *P* = 0.012] and LDL-C [OR: 1.892 (95% CI: 1.106–3.239), *P* = 0.020] were significant predictors of TCFA.

**Conclusions:**

High level of PLR may be associated with vulnerable plaque features of non-culprit lesions in patients with ACS. PLR, a cheap and easily available index, may surve as a useful inflammatory marker in reflecting plaque vulnerability.

## Background

Inflammation plays a vital role in the pathophysiological process of atherosclerotic disease [1]. At the instigation of atherogenic diet, endothelial cells became inflamed and proceeded with attracting leukocyte to the nascent atherosclerotic position. As the evolution of atherosclerotic lesions, leukocytes and other vascular wall cells secrete various proinflammatory mediators, which subsequently render the plaque vulnerable and prone to rupture. Vulnerable plaque, characterized by thin fibrous caps, large lipid core, macrophage infiltration and neovascularization, is closely related to inflammation [2–4]. The majority of acute coronary syndromes (ACS) can be attributed to plaque vulnerability [5].

Previous researches have demonstrated that elevated platelet counts or reduced lymphocyte counts were related to poor cardiovascular clinical outcomes [6–10]. Furthermore, platelet to lymphocyte ratio (PLR), initially served as a systemic inflammatory biomarker to predict the prognosis of neoplastic diseases [11–13], recently showed distinct predictive value on mortality or major adverse cardiovascular events [14, 15]. The severity of coronary artery disease was likewise shown to be associated with PLR [16, 17]. In spite of this, the relationship between PLR and atherosclerotic plaque vulnerability is still unclear.

Optical coherence tomography (OCT) has advantages in differentiating the vulnerable plaque including thin-cap fibroatheroma (TCFA) [18]. On account of high resolution, OCT made it possible to visualize the microstructure of vulnerable plaque in either culprit or non-culprit lesions in ACS [19]. Based on the above analyses, we aimed to evaluate whether high preoperative PLR was related to plaque vulnerability of non-culprit lesions in patients with ACS.

## Methods

### Study population

The present retrospective study included seventy-one patients who were diagnosed with ACS (non-ST segment elevated ACS and ST segment elevated myocardial infarction) and received percutaneous coronary intervention during admission between October 2012 and January 2014. Patients with chest pain that persisted for at least 30 min, ST-segment elevation > 1 mm in at least 2 contiguous leads or new-onset left bundle branch block on a 12-lead electrocardiogram, and elevated cardiac markers such as creatine kinase-myocardial band or troponin T/I were diagnosed with ST-segment elevated myocardial infarction (STEMI). The definition of non-ST segment elevated ACS included unstable angina pectoris (UAP) and non-ST segment elevated myocardial infarction (Non-STEMI). We defined non-STEMI as an acute myocardial infarction in the absence of elevated ST-segment on electrocardiogram. UAP was defined as new-onset angina, accelerated angina or angina at rest episodes but without cardiac markers elevation. In the present study, we defined non-culprit lesions as de novo atherosclerotic lesions with an angiographically intermediate diameter stenosis (50% to 75%) in the non-culprit/non-target locations. All of the patients had written informed consent prior to the enrollment. Our study was approved by the ethics committee at the Second Affiliated Hospital of Harbin Medical University (Harbin, China).

The exclusion criteria of this study comprised left main diseases, ostial lesions, severely calcified or tortuous lesions, left ventricular ejection fraction < 40%, cardiogenic shock, renal insufficiency (baseline serum creatinine > 2.0 mg/dl), or accompanied with malignant disease, peripheral arterial disease, chronic obstructive lung disease, hematologic disease, autoimmune disease, or other systemic inflammatory conditions.

### Laboratory tests

For all patients, venous blood samples were drawn from antecubital vein immediately after admission. The parameters of differential leukocyte count and platelet count were determined. Glycosylated hemoglobin, lipid profiles, creatinine and high sensitivity C-reactive protein (hs-CRP) were also assessed. The PLR was calculated as the ratio of platelet count to lymphocyte count and the neutrophil-to-lymphocyte ratio (NLR) was calculated by dividing neutrophil count by lymphocyte count.

### Angiographic analysis

The lesion distribution and plaque location of patients were recorded. Quantitative coronary angiography (QCA) was analyzed using the off-line software (CAAS 5.10.1, Pie Medical Imaging BV, Maastricht, the Netherlands). The reference vessel diameter (RVD), minimum lumen diameter (MLD) and diameter stenosis (DS) were analyzed.

### OCT image acquisition and analysis

**Fig. 1:**
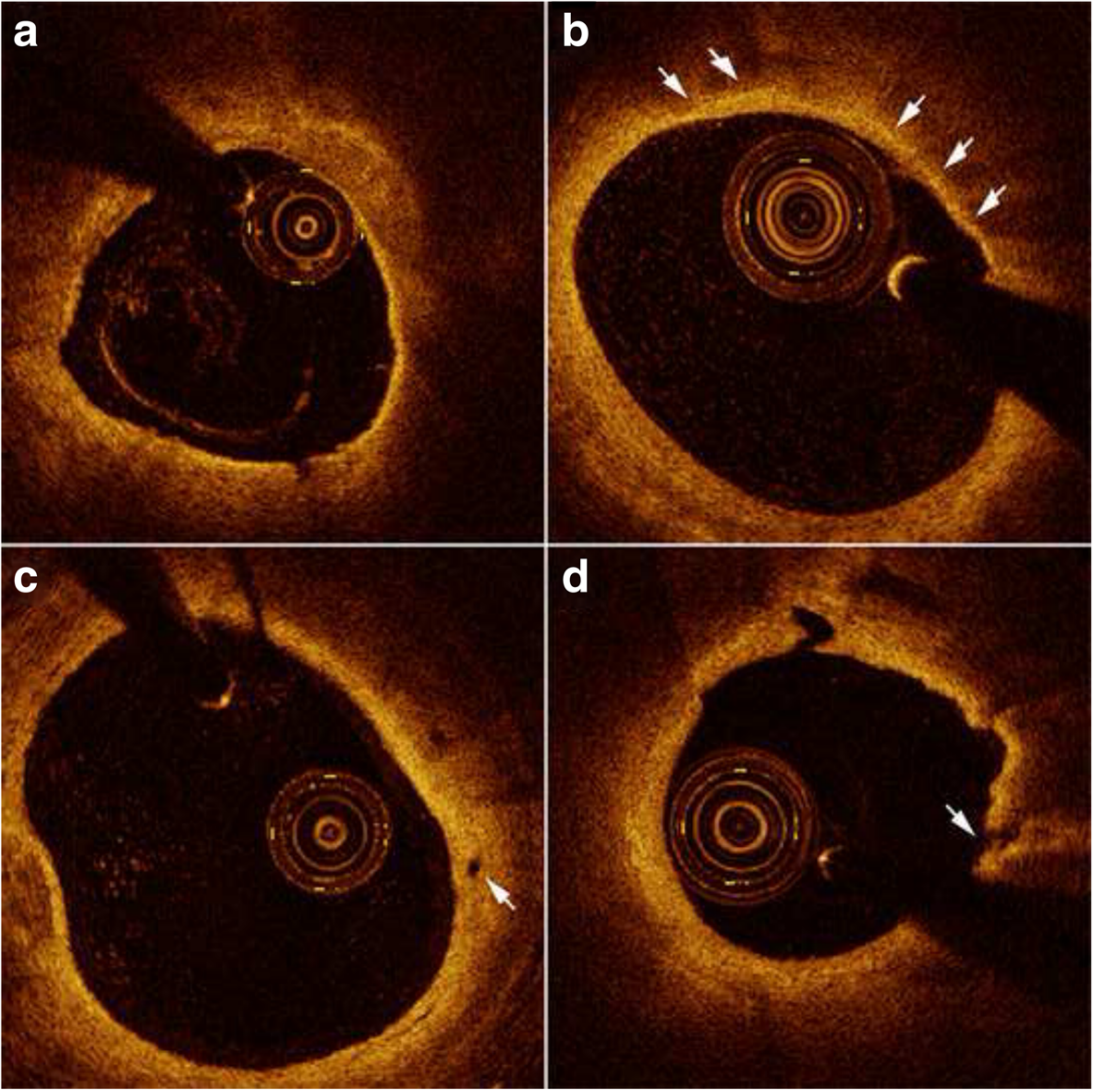
Representative optical coherence tomography images. **a** Thin cap fibroatheroma (TCFA) was displayed as a lipid-rich plaque (maximum lipid core > 90°) with fibrou cap thickness < 65 μm. **b** Macrophage infiltration (*arrows*) was defined as clusters of bright spots with backward shadowing. **c** Microchannel (*arrow*) was shown as a black hole in the plaque. **d** Ruptured plaque (*arrow*) was observed as a plaque with the discontinuous fibrous cap and a communication between the lipid core and the lumen

Intracoronary OCT examination was performed by using frequency-domain OCT system and the procedure was conducted as previously described [20]. Two independent observers, blinded to angiographic and laboratory characteristics, analyzed the OCT images according to the criteria for plaque measurement [21]. A signal-poor region with unclearly delineated borders was identified as a lipid core. The fibrous cap on OCT images appeared as signal-rich homogeneous regions overlying the lipid core. The fibrous cap thickness (FCT) was measured 3 times at the thinnest part of fibrous cap and the average was recorded. The maximum lipid arc was measured likewise on the cross-sectional images. When a lipid core took up at least two quadrants (maximum lipid core > 90°), it was defined as a lipid-rich plaque. Thin-cap fibroatheroma (TCFA) was defined as a lipid-rich plaque with the thinnest FCT < 65 μm (Fig. 1a). Calcification on OCT images appeared as a sharply delineated, heterogeneous region. Macrophage infiltration was defined as clusters of signal-rich spots in fibrous cap with backward shadowing (Fig. 1b). Cholesterol crystals were identified as linear regions with high signal intensity in the plaque. The presence of a tubular structure with the diameter of 50-300 μm on more than 3 consecutive cross sections was considered as a microchannel (Fig. 1c). Plaque rupture was defined as a plaque with the fibrous cap discontinuity and a cavity formation within the plaque (Fig. 1d). Plaque erosion was defined as formation of thrombus adjacent to the plaque surface without signs of overlying fibrous cap discontinuity. Thrombus on OCT images appeared as an irregular mass protruding into the lumen or attached to luminal surface.

### Statistical analysis

Continuous variables are expressed as mean ± standard deviation (SD) or median (25th to 75th percentiles), according to the distribution type of variables. Categorical variables are expressed as number (percentages). Continuous values were compared by independent sample *t*-test or Mann-Whitney *U* test. Categorical data were analyzed by chi-square or Fisher exact test. Correlations between PLR and plaque characteristics were assessed using Pearson correlation test or Spearman correlation rank test. To determine the important factors which indicate the presence of TCFA, univariate and multivariate logistic regression analysis were performed. Parameters with *P* < 0.05 in the univariate analysis were entered into the multivariate analysis models. A 2-tailed *P* value < 0.05 was considered statistically significant. All of the statistical analyses were performed by IBM SPSS version 19.0 (IBM Corp., Armonk, NY, USA).

## Results

### Baseline characteristics

Seventy-one patients with 119 non-culprit plaques were enrolled in this study. According to the median of PLR (109), enrolled patients were divided into low PLR group (PLR < 109, 36 patients with 69 plaques) and high PLR group (PLR > 109, 35 patients with 50 plaques). The baseline clinical characteristics and laboratory parameters of the two groups are shown in Tables 1 and 2. The prevalence of dyslipidemia was higher in low PLR group (41.7% vs. 20.0%, *P* = 0.048), whereas the percentage of renin-angiotensin system (RAS) blocker usage was lower in low PLR group (19.4% vs. 45.7%, *P* = 0.018). No other differences were observed between the two groups.

**Table 1:** Baseline Characteristics

Parameters	Low PLR (*n* = 36)	High PLR (*n* = 35)	*P* Value
Age, years	57.97 ± 10.51	59.77 ± 8.88	0.439
Male, n (%)	28(77.8%)	26(74.3%)	0.73
Type of ACS, n (%)			
UAP	29(80.6%)	27(77.1%)	0.725
Non-STEMI	4(11.1%)	4(11.4%)	0.966
STEMI	3(8.3%)	4(11.4%)	0.662
Hypertension, n (%)	23(63.9%)	17(48.6%)	0.193
Diabetes mellitus, n (%)	13(36.1%)	13(37.1%)	0.928
Dyslipidemia, n (%)	15(41.7%)	7(20.0%)	0.048
Current smoker, n (%)	15(41.7%)	8(22.9%)	0.09
Previous MI, n (%)	12(33.3%)	11(31.4%)	0.864
LVEF, %	0.65 ± 0.08	0.62 ± 0.08	0.262
Medical therapy			
ASA, n (%)	36(100.0%)	35(100.0%)	1.000
P2Y12 receptor antagonist, n (%)	34(94.4%)	35(100.0%)	0.493
Statin, n (%)	36(100.0%)	35(100.0%)	1.000
RAS blocker, n (%)	7(19.4%)	16(45.7%)	0.018
β-blocker, n (%)	15(45.7%)	17(48.6%)	0.559
CCB, n (%)	16(44.4%)	11(31.4%)	0.259

Values are mean ± SD or n (%)

*Abbreviations*: *PLR* platelet to lymphocyte ratio, *ACS* acute coronary syndrome, *UAP* unstable angina pectoris, *STEMI* ST-segment elevation myocardial infarction, *MI* myocardial infarction, *LVEF* left ventricular ejection fraction, *ASA* acetylsalicylic acid, *RAS* renin-angiotensin system, *CCB* calcium channel blocker

**Table 2:** Laboratory Parameters

Parameters	Low PLR (*n* = 36)	High PLR (*n* = 35)	*P* Value
Lymphocyte, ×10^9^/L	2.23 ± 0.45	1.66 ± 0.51	<0.001
Neutrophil, ×10^9^/L	4.80 ± 1.34	4.85 ± 2.23	0.898
Platelets, ×10^9^/L	202.75 ± 43.57	230.89 ± 45.47	0.01
NLR	2.21 ± 0.70	3.33 ± 2.31	0.007
RBC, ×10^12^/L	4.67 ± 0.49	4.54 ± 0.47	0.252
Hemoglobin, g/L	145.89 ± 16.65	139.46 ± 13.86	0.082
RDW, %	12.81 ± 0.55	12.97 ± 0.79	0.329
hs-CRP, mg/L	2.93 ± 4.04	3.03 ± 4.93	0.924
Hemoglobin A1c, %	6.53 ± 1.24	6.43 ± 1.49	0.797
Total cholesterol, mg/dl	167.95 ± 46.49	161.20 ± 38.33	0.507
LDL-C, mg/dl	91.58 ± 30.68	89.35 ± 31.06	0.762
HDL-C, mg/dl	47.89 ± 9.48	50.41 ± 14.09	0.382
Triglycerides, mg/dl	176.99 ± 104.43	128.62 ± 79.55	0.032
apoA, mg/dl	127.20 ± 22.27	134.11 ± 27.99	0.257
apoB, mg/dl	87.46 ± 33.45	79.77 ± 24.40	0.276
Creatinine, mg/dl	0.97 ± 0.22	0.95 ± 0.16	0.739
Troponin I, μg/l	1.77 ± 7.15	2.26 ± 7.12	0.774

Values are mean ± SD

*Abbreviations*: *NLR* neutrophil-to-lymphocyte ratio, *RDW* red cell distribution width, *CRP* C-reactive protein, *HDL-C* high-density lipoprotein cholesterol, *LDL-C* low-density lipoprotein cholesterol

As shown in Table 2, among laboratory parameters, the platelet number and NLR were significantly higher (*P* = 0.01 and *P* = 0.007, respectively), whereas the lymphocyte count was obviously lower (*P* < 0.001) in high PLR group. Meanwhile, the high PLR group presented with a mildly lower triglyceride level compared with the low PLR group (*P* = 0.032).

### Angiographic fingdings

The qualitative and quantitative characteristics of angiographic fingdings are listed in Table 3. There was no difference in the lesion distribution between the two groups. Meanwhile, we found no difference in RVD, MLD and DS between the low PLR and the high PLR group.

**Table 3:** Angiographic Findings

Parameters	Low PLR (*n* = 36)	High PLR (*n* = 35)	*P* Value
Total plaque number	69	50	
Vessel			0.159
LAD	26(37.7%)	18(36.0%)	
LCX	20(29.0%)	8(16.0%)	
RCA	23(33.3%)	24(48.0%)	
Lesion location			0.419
Proximal	26(37.7%)	15(30.0%)	
Mid	21(30.4%)	21(42.0%)	
Distal	22(31.9%)	14(28.0%)	
QCA data			
RVD, mm	3.36 ± 0.44	3.46 ± 0.51	0.253
MLD, mm	1.99 ± 0.58	2.10 ± 0.59	0.331
DS, %	41 ± 14	39 ± 15	0.576

Values are n (%) or mean ± SD

*Abbreviations*: *LAD* left anterior descending coronary artery, *LCX* left circumflex coronary artery, *RCA* right coronary artery, *QCA* quantitative coronary angiography, *RVD* reference vessel diameter, *MLD* minimum lumen diameter, *DS* diameter stenosis

### OCT findings

Table 4 shows OCT findings of the two groups. Patients in the high PLR group presented more frequent occurrence of TCFA than those in the low PLR group (34.0% vs. 15.9%, *P* = 0.022). FCT in the high PLR group was strikingly thinner (88.60 ± 44.70 vs. 119.28 ± 50.22 μm, *P* = 0.001), in addition, the high PLR group showed greater maximum lipid arc than the low PLR group (271.73 ± 71.66 vs. 240.60 ± 76.69°, *P* = 0.027).

**Table 4:** OCT Findings

Parameters	Low PLR (*n* = 36)	High PLR (*n* = 35)	*P* Value
Total plaque number	69	50	
Lesion length, mm	19.57 ± 7.94	20.88 ± 9.36	0.465
MLA, mm^2^	3.41 ± 2.01	3.77 ± 2.03	0.342
Maximum lipid arc, °	240.60 ± 76.69	271.73 ± 71.66	0.027
FCT, μm	119.28 ± 50.22	88.60 ± 44.70	0.001
TCFA, %	11(15.9%)	17(34.0%)	0.022
Macrophage, %	38(55.1%)	23(46.0%)	0.328
Calcification, %	32(46.4%)	25(50.0%)	0.696
Cholesterol crystal, %	7(10.1%)	6(12.0%)	0.749
Microvessel, %	26(37.7%)	21(42.0%)	0.634
Plaque rupture, %	9(13.0%)	3(6.0%)	0.208
Plaque erosion, %	0	2(4.0%)	0.094
Thrombus, %	9(13.0%)	5(10.0%)	0.611

Values are mean ± SD or n (%)

*Abbreviations*: *FCT* fibrous cap thickness, *TCFA* thin-cap fibroatheroma, *MLA* minimum lumen area

### PLR and OCT findings

**Fig. 2:**
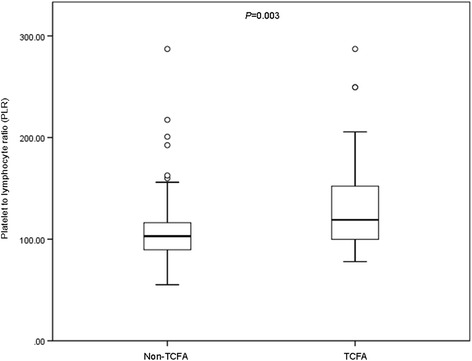
Comparison of PLR levels between patients with and without TCFA. The PLR value of the TCFA group (22 patients) was significantly greater than the non-TCFA group (49 patients) (*P* = 0.003)

**Fig. 3:**
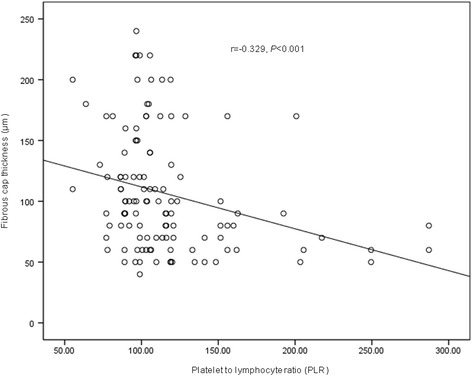
Correlation analysis of plate-to-lymphocyte ratio (PLR) and fibrous cap thickness (FCT). The PLR was negatively associated with FCT (*r* = −0.329, *P* < 0.001)

According to whether presenting with TCFA in non-culprit lesions, we divided study population into TCFA group (22 patients with 28 plaques) and non-TCFA group (49 patients with 91 plaques) and compared the PLR values between them. As presented in Fig. 2, the TCFA group showed significantly greater PLR than the non-TCFA group (*P* = 0.003). As shown in Fig. 3, there was a significant negative correlation between PLR and FCT (*r* = −0.329, *P* < 0.001).

We adopted univariate and multivariate logistic regression analysis to discuss which factors could predict the incidence of TCFA in non-culprit lesions of ACS (Table 5). Univariate regression analysis showed that LDL-C, PLR and NLR were possible predictors of the presence of TCFA. In multivariate regression analysis, LDL-C [odds ratio (OR): 1.892 (95% confidence interval (CI): 1.106–3.239), *P* = 0.020], PLR [OR: 1.023 (95% CI: 1.005–1.041), *P* = 0.012] remained independently predictable for TCFA.

**Table 5:** Logistic Regression Analysis of TCFA

Variables	Univariate		Multivariate	
	OR (95%CI)	*P* Value	OR (95%CI)	*P* Value
Age	1.004(0.962–1.047)	0.865		
Gender (male)	0.595(0.233–1.516)	0.276		
Diabetes mellitus	1.024(0.423–2.481)	0.958		
Current smoking	1.123(0.451–2.794)	0.803		
Prior MI	0.509(0.196–1.321)	0.165		
LDL-C	1.757(1.050–2.939)	0.032	1.892(1.106–3.239)	0.020
hs-CRP	0.996(0.906–1.095)	0.933		
PLR	1.015(1.005–1.025)	0.005	1.023(1.005–1.041)	0.012
NLR	1.242(1.001–1.539)	0.048	0.830(0.567–1.217)	0.341

## Discussion

The main findings of this study are as follows: (i) non-culprit plaques in ACS patients with higher PLR values exhibited more vulnerable characteristics (thinner FCT, greater maximum lipid arc and higher incidence of OCT-detected TCFA); (ii) PLR values were significantly and negatively correlated with FCT of non-culprit plaques; (iii) in multivariate regression analysis, PLR manifested as an independent indicator of TCFA in non-culprit leisons. To the best of our knowledge, this is the first study assessing the relation between PLR values and non-culprit plaque vulnerability using OCT in ACS patients.

TCFA has been postulated to be the precursor lesion of plaque rupture and subsequent luminal thrombosis which is the leading cause of ACS [5, 22, 23]. In ACS patients, besides of culprit lesions, the non-culprit lesions are often characterised by TCFA. A previous intravascular ultrasound (IVUS) sutdy demonstrated that ACS patients possessed more IVUS-derived TCFA in non-culprit lesions comparing with stable patients [24]. In another study, Kato et al. [25] evaluated the plaque characteristics of non-culprit leisons in patients with and without ACS by means of OCT, the results showed that TCFA was more frequent in the non-culprit lesions of ACS patients (64.7% versus 14.9%). However, in our present study, the OCT-detected TCFA in non-culprit lesions was fewer (31.0%). This discordance perhaps due to the relatively fewer incidence of plaque rupture (14.1%) in our study. Vergallo et al. [26] previously showed that patients with non-culprit rupture presented with higher frequency of TCFA.

PLR has recently been demonstrated to be correlated with various cardiovascular diseases [27–30]. In patients with acute myocardial infarction, PLR was also an independent predictor of short-term and long-term adverse clinical events [14, 15]. In addition, Kurtul et al. [16] demonstrated that higher PLR values (PLR ≥ 116) on admission were positively associated with the intermediate to high SYNTAX score in patients with ACS. Even so, the relationship between PLR and plaque characteristics has not been directly explored. By virtue of OCT which has advantages in identifying vulnerable plaques in vivo, our study provided the direct association of PLR and non-culprit plaques vulnerability in ACS patients. The mechanisms of the link between PLR and plaque vulnerability may be associated with immuno-inflammatory response which plays a crucial role in the plaque destabilization [31]. Previous studies demonstrated that PLR was positively associated with systemic inflammation markers such as CRP [16, 17]. Furthermore, higher platelet or lower lymphocyte counts per se can be regarded as the response to inflammatory stimuli [32, 33]. In ACS patients, platelet-derived chemokines, such as CXCL4 and CCL5, were elevated and played an important role in mediating the inflammatory response to plaque destabilization [34]. On the other hand, lymphocytopenia was also a pervasive phenomenon in the setting of ACS, which may be associated with lymphocytes apoptosis induced by inflammation [35].

In accordance with our findings, the ACS patients in the high PLR group possessed higher NLR values likewise (Table 2). Meanwhile, we found that the PLR was significantly correlated with NLR (*r* = 0.463, *P* < 0.001) which has been well-defined as a predictive marker for coronary artery disease severity. Arbel et al. [36] found that higher NLR values (NLR > 3) were associated with more serious coronary artery disease and worse prognosis. Nilsson et al. [37] showed that NLR was significantly associated with non-calcified plaques detected by coronary computed tomographic angiography. The combined usefulness of PLR and NLR in predicting adverse clinical events in patients with CAD was also demonstrated by Cho et al. [38], their findings showed that higher preoperative PLR and NLR, alone or combined, were significant predictors of long-term adverse clinical events. Further studies will be needed to identify the combined usefulness of PLR and NLR in predicting atherosclerotic plaque vulnerability.

It has been verified that the majority of ACS patients were at a high risk of recurrent cardiac events due to the lesions irrelevant to the initial ischemic events [39, 40]. According to the findings of PROSPECT study [41], non-culprit lesions and culprit lesions at baseline were equally contributed to the recurrence of major adverse cardiovascular events, furthermore, the non-culprit lesions associated with recurrent events were more likely to be IVUS-detected TCFA. In our study, the PLR presented as an independent indicator of OCT-detected TCFA in non-culprit lesions of ACS patients in multivariate regression analysis. We speculated that PLR, a simple and immediately obtained parameter, may be useful in identifying vulnerable plaques and patients with high risks. However, further studies are needed to verify all of our speculations.

### Limitations

There are several limitations in the present study. Firstly, the sample size in the current study was small, so more well-designed studies are warranted to confirm our findings. Secondly, this is a retrospective, single-central, cross-sectional study with a specific patient cohort, therefore we should be cautious to generalize the results to all patients. Thirdly, instead of assessing dynamic changes, we just evaluated the spot value of PLR so that we cannot ensure whether it remains an indicator of TCFA subsequently. Finally, because of the limited penetration of OCT, we were not able to show additional characteristics related to plaque vulnerability (for example plaque burden).

## Conclusions

The OCT-detected TCFA in non-culprit lesions is more frequent in ACS patients with higher levels of PLR. Higher PLR levels show an important indicator of TCFA in non-culprit lesions. These findings reflect that PLR may serve as a useful indicator of atherosclerotic plaque vulnerability. Future prospective studies with large-scale samples are expected to validate the findings in our study.

## Abbreviations

ACS: Acute coronary syndrome
DS: Diameter stenosis
FCT: Fibrous cap thickness
hs-CRP: High sensitivity C-reacitve protein
IVUS: Intravascular ultrasound
MLD: Minimum lumen diameter
NLR: Neutrophil-to-lymphocyte ratio
OCT: Optical coherence tomography
PLR: Platelet to lymphocyte ratio
QCA: Quantitative coronary angiography
RVD: Reference vessel diameter
STEMI: ST-segment elevated myocardial infarction
TCFA: Thin-cap fibroatheroma
UAP: Unstable angina pectoris.
